# Exome Sequencing uncovers Homozygous Stop-Gained variant in the *SYNE1* Gene Leading to Spinocerebellar Ataxia

**DOI:** 10.12669/pjms.42.7.15376

**Published:** 2026-07

**Authors:** Absarul Haque, Mohammad Zubair Alam, Fehmida Bibi, Osama Yousef Muthaffar, Mahmood Rasool, Muhammad Imran Naseer

**Affiliations:** 1 Absarul Haque King Fahd Medical Research Centre, Department of Medical Laboratory Technology, Faculty of Applied Medical Sciences, King Abdulaziz University, Jeddah, Saudi Arabia; 2 Mohammad Zubair Alam King Fahd Medical Research Centre, Department of Medical Laboratory Technology, Faculty of Applied Medical Sciences, King Abdulaziz University, Jeddah, Saudi Arabia; 3 Fehmida Bibi King Fahd Medical Research Centre, Special Infectious Agents Unit–BSL3, King Abdulaziz University, Jeddah, Saudi Arabia; 4 Osama Yousef Muthaffar Department of Pediatrics, Faculty of Medicine, King Abdulaziz University, Jeddah, Saudi Arabia; 5 Mahmood Rasool Institute of Genomic Medicine Sciences (IGMS), Department of Medical Laboratory Technology, Faculty of Applied Medical Sciences, King Abdulaziz University, Jeddah, Saudi Arabia; 6 Muhammad Imran Naseer Institute of Genomic Medicine Sciences (IGMS), Department of Medical Laboratory Technology, Faculty of Applied Medical Sciences, King Abdulaziz University, Jeddah, Saudi Arabia

**Keywords:** SYNE1, ARCA1 and SCAR8, Ataxia, Saudi Arabia

## Abstract

**Background &Objective:**

Spectrin Repeat-Containing Nuclear Envelope Protein 1 (SYNE1) is important gene for maintaining neuronal structure and function, particularly in the cerebellum, the brain region responsible for coordinating movement. The genetic mutation in *SYNE1* gene, which encodes Nesprin-1 protein leads to autosomal recessive form of spinocerebellar Ataxia OMIM (608441). This cerebellar dysfunction causes progressive balance and coordination problems, including reflexes and cognitive impairment. To understand genetic mutations in *SYNE1* gene that are linked with Autosomal Recessive Spinocerebellar Ataxia type 8 (SCAR8) and Autosomal Recessive Cerebellar Ataxia type 1 (ARCA1).

**Methodology:**

The study was done in the Center of Excellence in Genomic Medicine and Research (CEGMR) during 2023-2024. Firstly, Whole Exome Sequencing (WES) was carried out to identify the mutation, followed by Sanger sequencing to validate the WES results.

**Results:**

WES identified a novel homozygous stop-gained variant, NM_182961.3:c.352C>T (p.Arg118Ter), in the *SYNE1* gene in a 32-year-old Saudi patient. This alteration was associated with progressive cerebellar atrophy, impaired fine motor skills, muscular weakness, and speech and learning deficits. The variant was independently confirmed by Sanger sequencing.

**Conclusions:**

The patient’s phenotype was consistent with previously reported ARCA1 and SCAR8. To our knowledge and based on the currently available literature, this may represent the first reported Saudi family with a *SYNE1* mutation associated with these conditions. This finding advances the genetic and molecular characterization of these rare disorders, highlights the utility of molecular diagnostics, and supports the establishment of a local database of disease-associated variants to improve diagnosis, management, and future research in the Saudi population.

## INTRODUCTION

Autosomal recessive spinocerebellar ataxias (ARCA or SCAR) are considered as a sizeable, and extremely heterogeneous group of neurodegenerative disorders.^1^ Autosomal recessive disorder is now uniformly referred to as Spinocerebellar Ataxia, Autosomal Recessive 8 (SCAR8), previously known as ARCA1, is a rare neurodegenerative disorder caused often characterised as the central and peripheral nervous systems caused by mutations in the *SYNE1* gene.^2^ Friedreich ataxia (FA), is the most frequent autosomal recessive ataxias include ataxia with vitamin E deficiency, ataxia telangiectasia, autosomal recessive spastic ataxia of Charlevoix-Saguenay (ARSACS) along with abetalipoproteinemia, ataxia with oculomotor apraxia (AOA) are associated with a complex phenotype.^3^

ARCA1 is characterized by marked phenotypic heterogeneity, with manifestations ranging from cerebellar dysfunction to auditory, neuropathic, ophthalmologic, skeletal, cutaneous, and cognitive abnormalities.^4^ Usually, diagnosis of ARCA1 remains challenging because of their low prevalence, poor medical recognition, and heterogeneous clinical presentation with many overlapping features between entities.^3^ So, it relies on integrated approach, combining detailed clinical assessment, neuroimaging (MRI), and genetic assessment, while current management is primarily symptomatic due to the lack of curative therapy.^3^

Recent advances in ARCA1 genetics, driven by advanced next generation sequencing technologies such as whole exome and whole genome sequencing, have improved diagnostic accuracy by identifying molecular causes in many previously undiagnosed, clinically heterogeneous ataxias.^5^ These approaches also refine genotype frequencies and reveal novel phenotypes, expanding molecular understanding, including Friedreich ataxia and RFC1-related ataxia.^5^

Numerous recent studies have established that the mutations in the spectrin repeat-containing nuclear envelope 1, *SYNE1* gene is the main causative agent for Autosomal recessive spinocerebellar ataxia type 8 (ARCA1/SCAR8) disorder 6. SYNE1, one of the largest human genes, comprising 146 exons and encodes Nesprin-1 (nuclear envelope spectrin 1), an 8797-amino-acid spectrin family structural protein linking the plasma membrane to the actin cytoskeleton.^7^ Late-onset, slowly progressive cerebellar ataxia with dysarthria, but without dysmetria, was reported in 26 French Canadian families from Quebec, involving 53 affected individuals.^8^

A severe SCAR8 variant was also reported in a Japanese woman from consanguineous parents.^9^ Additionally, 23 European patients with SCAR8 were described, 22 of whom had biallelic truncating alleles, while one had a compound heterozygous missense and truncating allele.^7^ Recently, we reported a novel homozygous PMPCA variant (NM_015160.2: c.802C>T p.(Arg268Trp)) associated with SCAR type 2.^10^ Another study reported SCAR17 associated with a novel ATM gene mutation in a female patient with ataxia telangiectasia.^10^,^11^ Moreover, episodic ataxia type 2 (EA2) is a heterogeneous neurological disorder caused by CACNA1A gene mutations, and a rare SETX gene mutation has recently been reported in a Saudi patient.^12^,^13^ Additionally, GBA2 mutations have been implicated in ARCA with spasticity.^14^

SCAR8, a rare neurodegenerative disorder, has not been previously reported in Saudi Arabia. However, this study uncovers a novel homozygous stop-gain variant in the *SYNE1* gene in a Saudi male with progressive cerebellar ataxia. This discovery not only deepens our understanding of *SYNE1* gene related disorders but also sheds light on the mechanisms behind SCAR8 in the Saudi population. With further validation in larger cohorts, this variant could become a potential molecular diagnostic biomarker.

## METHODOLOGY

This study included a 28-year-old male proband presenting with progressive cerebellar ataxia characterized by impaired coordination, gait instability, abnormal balance, reduced reflexes, and deteriorating motor skills. The patient also exhibited learning difficulties without evidence of intellectual deterioration or dysmorphic features. Neurological examination revealed cerebellar dysfunction, while ocular movements and cranial nerve functions were preserved. Brain MRI findings were evaluated as part of the clinical workup. Due to the progressive neurological presentation and suspected hereditary etiology, molecular genetic testing was performed to identify the underlying genetic cause.

### Ethical statement:

The study was approved by the ethical committee (Ref# 013-CEGMR-1-ETH, dated: 1, May 2013) of the Center of Excellence in Genomic Medicine Research, King Abdulaziz University, Jeddah. This study was done in CEGMR KAU, during 2023-2024. Written informed consent of patients was obtained prior to genetic analysis, following the Declaration of Helsinki (2013) and international ethical guidelines.

### Inclusion and exclusion criteria:

The study included patients with clinical features of hereditary cerebellar ataxia progressive balance impairment, coordination deficits, motor dysfunction, or related neurological signs requiring molecular evaluation; individuals lacking relevant phenotypes or sufficient clinical/genetic data were excluded.

### Sample collection:

Peripheral blood samples were collected for genomic DNA extraction. DNA quality and concentration were assessed using a NanoDrop™ 2000/2000c spectrophotometer.

### Whole exome sequencing (WES):

WES was performed to analyze protein-coding regions (exons) and non-coding region (splice-site junctions) of the targeted genes. Sequencing was carried out on an Illumina NextSeq platform using 2 × 76 bp paired-end reads.^15^ Sequence data were aligned to the human reference genome hg19/GRCh37 (UCSC). Bioinformatics tools were utilized to predict disease-associated variants. All sequence alterations were annotated according to Human Genome Variation Society nomenclature guidelines.

After WES, FASTQ files were generated and subsequently converted into BAM and variant call format (VCF) files containing candidate genetic variants. Identification of targeted variants possibly leading to the disease phenotype was established through rare, ultra-rare, novel (MAF <0.01%) frequency, homozygosity or heterozygosity settings, genomic position, changes in structure and function (PolyPhen/SIFT used for damage prediction), protein damaging, pathogenicity etc. Moreover, multiple bioinformatics tools and filtering pipelines were used for analysis with GRCh37 as reference genome. Furthermore, identified variants were filtered against public databases, including Genome Aggregation Database (gnomAD), to assess allele frequencies below 5.0%. Moreover, Frameshift, nonsense, and splice-site variants in disease-associated genes with a minor allele frequency ≤1.0% were further evaluated by comparison with public databases, including gnomAD, dbSNP (https://www.ncbi.nlm.nih.gov/SNP), 1000 Genomes Project (http://www.internationalgenome.org).^14^,^16^,^17^ Notably, potential deleterious effects of the gene and its product, including evolutionary, splicing impacts, and conservation, were assessed using computational prediction tools. Identified variants were classified according to American College of Pathologists (ACMG/AMP) guidelines. Novel variants were documented following HGVS nomenclature (https://varnomen.hgvs.org/). In silico analysis evaluated deleterious effects on protein structure and function and their relationship to disease phenotype and functional defects.

### Sanger sequencing:

The WES results were validated by Sanger sequencing to confirm the novel homozygous SYNE1 variant. Primers were designed using Primer3 software, and Sanger sequencing was used to assess disease segregation in affected family members. Similarly, targeted primers were designed with forward primer SYNE1F: 5’-TGTTTGTTTCGGTGCCTCAC-3’ and reverse primer SYNE1R: 5’-CAAAGATATGGCAAATGAGAGC-3’. After PCR amplification, amplicons were purified and DNA quality and quantity were assessed using Nanodrop. Purified DNA was used for cycle sequencing using BigDye Terminator v3.1 kit with a single specific primer of the target DNA sequence. The reaction was performed in a thermal cycler following the manufacturer’s standard cycling protocol. After purification, samples were subjected to capillary electrophoresis using an Automated DNA sequencing machine (3500xL Genetic Analyzer) from Applied Biosystem. Raw data were analyzed for quality, and sequencing files were aligned with the reference sequence using BioEdit software to identify mutant variants.

### Protein structure predication of mutant SYNE1:

The SYNE1 sequence was retrieved from the UniProt database (UniProt ID: Q8NF91). Homology modeling of SYNE1 was conducted using the wild-type SYNE1 (AF ID: AF-F5H4Q0-F1-v4) as a template in Swiss-Model.^18^ The partial structure of wild-type SYNE1 was obtained from the AlphaFold database (ID: AF-F5H4Q0-F1),^18^ and the results were visualized using BIOVIA Discovery Studio visualizer.^19^

## RESULTS

SYNE1 Ataxia is a rare genetic neurological disorder triggered by mutations in the *SYNE1* gene, affecting balance and coordination. Although present from birth, symptoms typically appear between early childhood and mid-adulthood, often by age 30. It accounts for about 5% of all recessive Ataxia. In this study, WES results analysis led to identify a novel homozygous stop-gained variant, NM_182961.3: c.352C>T p.(Arg118Ter) in *SYNE1* gene. The patient sample showed a homozygous nonsense mutation in the *SYNE1* gene (c.352C>T; p.Arg118Ter), where arginine is replaced by a premature stop codon, leading to a truncated protein. The null variant in this gene, causing complete loss of function, is a known disease mechanism and is classified as pathogenic (PVS1) per ACMG/Franklin criteria; its extremely low frequency in gnomAD further supports classification under PM2.The identified novel homozygous stop-gained variant was further validated by computational approach, and the reported identical alleles variants (nonsense mutation) were again predicted to have unanimously deleterious effect on the gene function.

**Fig.1:**
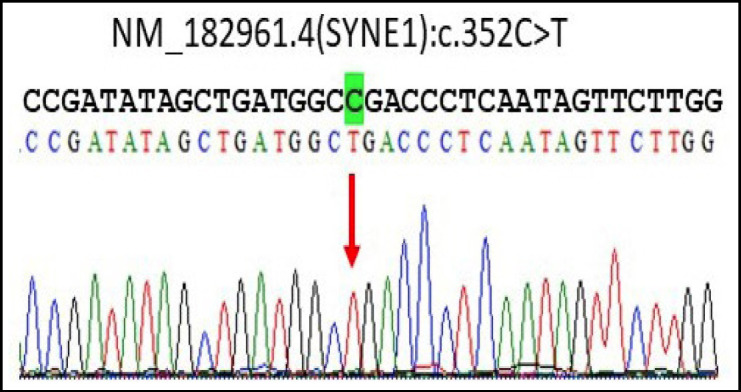
Representative electropherogram of SYNE1 gene c.352C>T. Sanger sequencing results showed change of “T” base pair were “C” base pair is in normal wild type condition.

This gene plays an important role by encoding a spectrin repeat containing protein, which is expressed in smooth and skeletal muscle, along with circulating lymphocytes, that localizes to the nuclear membrane protein belonging to the Nesprin family. Mutations in this gene are associated with autosomal recessive spinocerebellar ataxia 8 (also known as autosomal recessive cerebellar ataxia type 1). The patient, a 28-year-old male, is homozygous (T/T) as confirmed by Sanger sequencing (Fig.1). To exclude false positives, 100 healthy Saudi controls were screened by WES, confirming the absence of the identified variant in the normal population these controls represent previously sequenced and internally curated Saudi exomes, which were re-analyzed as a comparative dataset for variant validation. To the best of our knowledge, this novel homozygous variant has not been previously reported in this population; thus, we are the first to identify this SYNE1 mutation associated with autosomal recessive spinocerebellar ataxia 8 in Saudi patients. The variant is absent from gnomAD exomes and the 1000 Genomes database, indicating it is rare or novel and warrants further investigation for pathogenicity. It introduces a premature stop codon (Arg→Ter), resulting in a truncated protein and early termination of translation. We have clearly verified the variant *SYNE1* ataxia (NM_182961.3: c.352C>T; p.Arg118Ter) against major population databases, including gnomAD and ClinVar. The variant was found to be absent from both gnomAD exomes and genomes, as well as not previously reported in ClinVar, supporting its extreme rarity.

**Fig.2:**
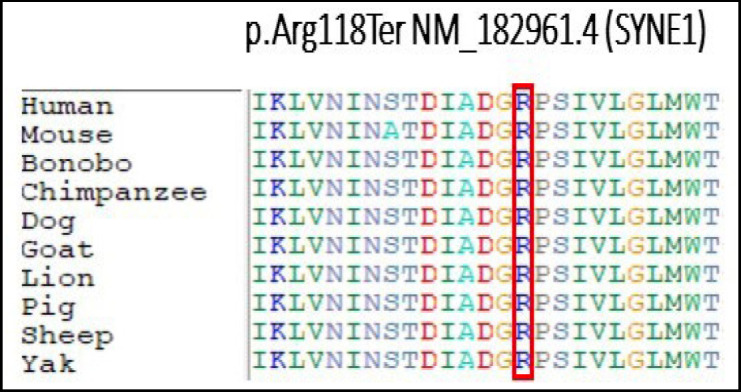
The amino acid arginine at residue 118 in human SYNE1 is highly conserved across many species, including Bonobo, mouse, Cow, Lion, Chimpanzee, Sheep, Dog and Goat.

**Fig.3:**
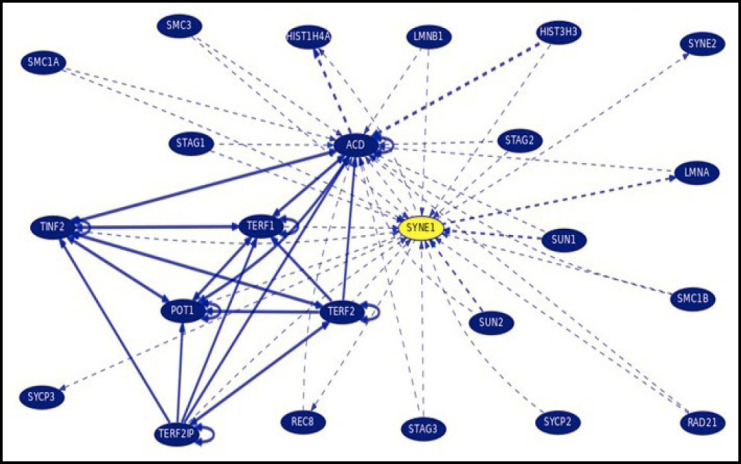
USCS SYNE1 Gene interactions and pathways from curated databases and text-mining.

In silico analyses using multiple computational tools suggest that the homozygous stop-gain mutation in *SYNE1* is deleterious, impacting evolutionary conservation, functional domains, and potentially splicing (Fig.2). The Arg→Ter substitution (Stop codon) occurs at a highly conserved residue across diverse species, indicating its functional importance. This premature stop codon leads to protein truncation and early termination of translation, likely abolishing normal protein function. Additionally, loss of the positively charged arginine residue may disrupt local electrostatic interactions and protein folding, contributing to structural instability and functional impairment. Moreover, UCSC gene interactions and pathways from curated databases and text-mining showed the interaction of the gene with other important genes as shown in (Fig.3).

**Fig.4:**
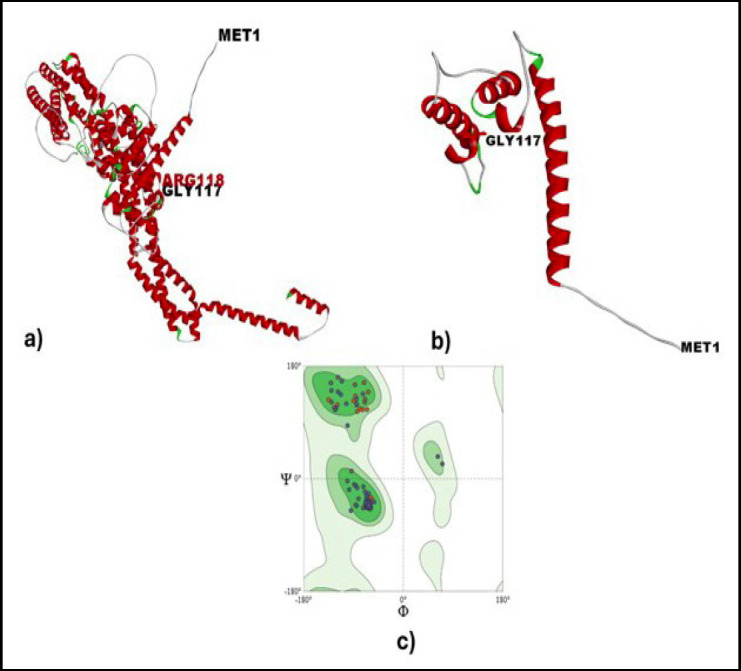
Structural analysis of the (Arg118Ter) variant. a), showing the wild type normal while b), showing the mutated protein and c). Ramachandran plot.

Homology modeling of mutant *SYNE1* (p.Arg118*) was performed using the wild-type structure (AF ID: AF-F5H4Q0-F1). The mutant model, generated via Swiss-Model and validated by Ramachandran analysis (Fig.4), showed 100% residues in favored regions. A MolProbity score of 0.50 and clash score of 0.00 indicate optimal stereochemistry and no significant atomic overlaps. These validation metrics substantiate the structural quality and reliability of the model. Notably, to date, the complete experimental determined structure of SYNE1 is not available in the Protein Data Bank (PDB), largely due to the protein’s considerable size, intrinsic flexibility, and membrane-associated localization, which present significant challenges for high-resolution structure determination by X-ray crystallography, nuclear magnetic resonance (NMR) spectroscopy, or cryo-electron microscopy (cryo-EM).

## DISCUSSION

WES was used to identify the genetic cause of spinocerebellar ataxia in this patient. It is a powerful, cost-effective diagnostic tool for rare genetic disorders, with a 30–50% yield in inherited ataxias, supporting its role as a first-line test in complex neurological cases.^20^,^21^

In the present study, a homozygous *SYNE1* variant (c.352C>T; p.(Arg118Ter) was identified, introducing a premature termination codon at position 8746 and early protein truncation. This stop-gain mutation in the final exon may significantly affect SYNE1 protein stability and function.^22^ Additionally, we identified a novel homozygous stop-gain variant (NM_182961.3: c.352C>T; p.(Arg118Ter)) in a 28-year-old Saudi patient with progressive cerebellar symptoms. WES enables simultaneous analysis of multiple ataxia-associated genes, facilitating the identification of pathogenic variants and improving the efficiency of molecular diagnosis. All WES findings were validated by Sanger sequencing, the gold standard for confirming nucleotide variants due to its high accuracy and reliability. Bidirectional sequencing confirmed the *SYNE1* variant (c.352C>T; p.(Arg118Ter)), supporting autosomal recessive inheritance with parental carrier status. Complete concordance between WES and Sanger sequencing ensured result reliability for clinical reporting and genetic counselling.^23^

To predict functional impact on protein structure and function, in silico analysis was performed. The pathogenicity of the novel *SYNE1* gene variant p.(Arg118Ter) was evaluated using SIFT, PolyPhen-2, and CADD scoring.^24^ A premature stop codon results in a severely truncated protein lacking key functional domains required for cytoskeletal organization and nuclear envelope integrity. Structural modeling suggests that p.(Arg118Ter) may trigger nonsense-mediated decay or produce a non-functional fragment of only 117 amino acids from the 8,797-residue SYNE1 protein, removing spectrin repeats, coiled-coil regions, and the C-terminal KASH domain required for nuclear envelope localization. Our identification of the p.Arg118* variant and SWISS-MODEL–based structural analysis aligns with current approaches for assessing functional consequences of genetic variants (Fig.4). SYNE1 nonsense mutations such as p.Arg118* introduce premature termination codons, producing truncated proteins and are implicated in ARCA Type-1.^25^ Structural modelling is widely used to predict phenotypic effects of non-synonymous SNPs in the absence of experimental data. Integrating 3D structural information improves mutation impact prediction accuracy, especially when evolutionary data is limited. Furthermore, recent studies emphasize the role of computational tools and homology modeling (e.g., SWISS-MODEL) in assessing SNP-related structural and functional impacts, supporting functional annotation of disease variants.^26^,^27^ Our combined approach of SNP identification and structural prediction is consistent with established methods for understanding SYNE1-related disease mechanisms.

*SYNE1* gene mutations are among the most common causes of ARCA and are associated with SCAR8 and ARCA1. It encodes Nesprin-1, a nuclear envelope scaffolding protein essential for nuclear positioning, cellular motility, and mechanotransduction. The novel variant c.352C>T (p.Arg118Ter) expands the mutational spectrum, with SCAR8-consistent clinical features in the Saudi patient.

As reported previously that *SYNE1* mutations are typically associated with slowly progressive cerebellar degeneration beginning in adolescence or early adulthood, consistent with the progressive cerebellar atrophy observed in our case. The absence of extrapyramidal symptoms further highlights the clinical heterogeneity of hereditary ataxias and the underlying pathophysiological diversity. Although our patient presented with slurred speech, learning difficulties, tremors, and impaired fine motor skills, nystagmus and intellectual disability were absent, which may relate to the normal cerebellar MRI findings, in contrast to previously reported cerebellar atrophy.

WES improves the detection of genetically heterogeneous disorders by enabling the identification of novel and causal variants, with diagnostic yields of 25–35%, and up to 40% in trio analysis. In addition, molecular diagnosis supports genetic counseling, prenatal testing, and family planning. In Saudi Arabia, consanguineous marriages significantly increase the incidence of autosomal recessive cerebellar ataxias, making comprehensive genetic testing particularly important for affected families. Molecular confirmation of SCAR8 variants facilitates cascade screening, informed reproductive decisions, and targeted genetic counseling, while also enabling access to emerging therapies and clinical trial opportunities.^28^,^29^

### Strength and Limitations:

The study’s strengths include the first identification of a novel homozygous *SYNE1* gene mutation causing SCAR8 in a Saudi patient, the use of genetic methods like WES and Sanger validation, detailed structural and computational analyses to understand mutation impact, clinical correlation of genetic findings, and emphasis on regional genetic data. Limitations involve being a single case report without functional experiments.

## CONCLUSION

This study reports the first case of SCAR8 in a Saudi family caused by a novel homozygous *SYNE1* gene variant (c.352C>T; p.(Arg118Ter)). The findings highlight the role of WES in identifying rare or novel variants and expanding the mutational spectrum of *SYNE1* gene in the Saudi population. We recommend routine use of WES in patients from consanguineous families with a strong history of hereditary ataxia. Further large-scale studies and functional analyses are needed to better define the *SYNE1* gene mutational landscape and disease pathophysiology.
